# Changes and their effects on working and daily life time use allocation between work-from-home and office work days during the telework period: insights from the survey in Japan

**DOI:** 10.3389/fsoc.2025.1534548

**Published:** 2025-02-04

**Authors:** Eri Aoki, Ai Hiramatsu, Keisuke Hanaki

**Affiliations:** ^1^Research Institute for Humanity and Nature, Kyoto, Japan; ^2^Faculty of Information Networking for Innovation and Design, Toyo University, Tokyo, Japan

**Keywords:** telework, work-from-home, time use allocation, questionnaire survey, Japan

## Abstract

**Introduction:**

The rapid adoption of telework, accelerated by advancements in ICT and the COVID-19 pandemic, offers potential benefits for wellbeing and environmental impact. However, telework’s effects on work productivity, work-life balance, and social connectedness remain complex, particularly within hybrid models combining work-from-home (WFH) and in office days.

**Methods:**

This study assessed telework’s impact by comparing WFH and office days. A survey of 1,500 full-time workers in Japan’s Tokyo Metropolitan Region focused on daily time allocation, and telework preferences during telework periods. Principal component and cluster analyses were used to identify groups with distinct work and lifestyle patterns.

**Results:**

Six telework-related groups emerged, reflecting diverse experiences in productivity and daily life. Groups such as the “Overall Increase” and “Housework and Rest Increase” reported gains in leisure and family time, positively impacting wellbeing. In contrast, the “Unsuitable for WFH” group faced increased office-day workloads and reduced WFH productivity, indicating that telework’s effectiveness depends on job and individual characteristics.

**Conclusion:**

The findings highlight telework’s potential to enhance wellbeing and sustainability but also underscore the need for tailored policies that address diverse job requirements and personal characteristics. This study contributes to sustainable telework strategies by offering insights into effective support systems that balance flexibility, productivity, and environmental sustainability, aiming both for an enhanced personal life and societal benefits.

## 1 Introduction

The development of information and communication technology, combined with the COVID-19 pandemic, has increased the feasibility of widespread telework adoption. Telework has the potential to become a new form of affluent living, contributing to decarbonization as an initiative that reduces environmental impact while improving economic activity and wellbeing. When considering the direct benefits of telework in the formation of a sustainable society, the primary advantage often cited is the reduction in traffic-related environmental impacts due to decreased commuting (Hook et al., 2020; Horner et al., 2016; O’Brien and Yazdani Aliabadi, 2020). In Japan, this has been highlighted since the government’s Kyoto Protocol Target Achievement Plan in 2008. The plan established a specific measure aimed at reducing traffic-related emissions by promoting “the use of telework and other information and communication technologies as alternatives to transportation, “with a target of reducing emissions by approximately 630,000 tons compared to 1990 levels by 2012. This target was achieved (Japan, 2008; Global Warming Solutions Implementation Headquarters, Government of Japan, 2014). Although no specific numerical targets were set for 2030 in the Global Warming Countermeasure Plan formulated by the government in October 2021, the plan continues to emphasize the promotion of these measures (Japan, 2021).

However, in the pursuit of a sustainable society, other noteworthy effects of telework should also be considered. By reducing geographical and time constraints, telework enables diverse groups to engage in meaningful work. Consequently, improvements in work-life balance, expansion of employment opportunities, and increased job satisfaction—social benefits that have been previously proposed—are critical in determining whether telework, as a new way of working, can be widely accepted by society (Beckel and Fisher, 2022; Laumer and Maier, 2021; Moglia et al., 2021). Additionally, telework has proven valuable in ensuring business continuity during emergencies, a role that was particularly evident during the recent COVID-19 pandemic (Belzunegui-Eraso and Erro-Garcés, 2020).

On the other hand, numerous negative aspects of telework have been reported, such as social isolation due to reduced communication, professional isolation which negatively impacts job performance and increases turnover intentions, declines in productivity, and increases in employee workload and stress (Bentley et al., 2016; Golden et al., 2008; Nemțeanu and Dabija, 2023). The impacts of telework can vary depending on the nature of the work and the individual situations, presenting both advantages and disadvantages (Ipsen et al., 2021; Mun et al., 2022; Vitória et al., 2022). In particular, during the COVID-19 state of emergency in 2020, workplaces that were suddenly forced into telework without sufficient preparation reported declines in both worker and family satisfaction, as well as in work productivity (Beno and Hvorecky, 2021; Morikawa, 2022). Additionally, concerns have been raised regarding telework’s effects on daily life, such as the blurring of boundaries between work and personal life and the increased burden of household chores (Tan et al., 2024; Tremblay et al., 2006). These findings suggest that balancing work and personal life, alongside effective time allocation, is key to enhancing individuals’ wellbeing.

The COVID-19 pandemic in 2020 led many people worldwide to adopt telework, creating an unintended social experiment where individuals experienced new ways of working and living, including changes in work-related communication, workplace dynamics, and work-life balance (Petcu et al., 2023; Rahmani et al., 2021; Watanabe et al., 2024). This shift provided an opportunity to reassess wellbeing, particularly the relationship between work and family life. As flexibility in work arrangements continues to expand and society progresses toward achieving the SDGs, a hybrid model of telework—combining both remote work and office attendance—is expected to become more prevalent. However, analyses focusing specifically on teleworkers and the balance between their work and personal lives remain limited. From the perspective of contributing to the SDGs, it is essential to analyze telework in a way that considers both the flexibility it offers and the diversity of its participants.

In this study, we analyzed the changes brought about by telework, considering not only the nature of work-from-home (WFH) days but also examining both WFH and office days during the telework period. Specifically, we conduct a systematic analysis of changes in workdays, work productivity, and time allocation between WFH and office days. The goal of this research is to clarify what types of support or policies are necessary for different types of people to promote telework as a work style that enhances productivity and wellbeing while reducing environmental impact throughout the telework period. By doing so, the study aims to provide valuable insights for the development of telework policies that support the transition to a sustainable society, ensuring that a diverse range of workers can benefit from telework.

## 2 Theoretical background

### 2.1 Prevalence of telework and its changes

The COVID-19 pandemic has significantly altered work patterns, particularly with the widespread adoption of telework, or working from home. Telework was rapidly embraced in many countries during the initial phase of the COVID-19 outbreak in 2020 (Alipour et al., 2021; Belzunegui-Eraso and Erro-Garcés, 2020; Kniffin et al., 2021). In the European Union, for example, 39% of the employed population began teleworking in April 2020 due to the pandemic, and by July 2020, 48% reported having teleworked at some point (Eurofound, 2020). In Japan, the government declared a state of emergency in April 2020 and strongly urged businesses to promote telework, with the goal of reducing the number of commuters by 70% (Novel Coronavirus Response Headquarters, the Government of Japan, 2020). While many companies returned to traditional commuting-based work styles in the latter half of 2020, a significant number—particularly in major metropolitan areas such as Tokyo—continued to implement telework systems. By 2021, the proportion of teleworkers among employed workers had risen to 27.0% nationwide, 42.1% in the Tokyo metropolitan area, and 17.7% in regional urban areas (Ministry of Land, Infrastructure, Transport and Tourism of Japan, 2022a). Furthermore, surveys conducted by the Ministry of Health, Labour and Welfare in 2020 (Mitsubishi UFJ Research and Consulting Co., Ltd., 2021) and the Ministry of Land, Infrastructure, Transport and Tourism of Japan (2022a) revealed that a large percentage of workers expressed a desire to continue teleworking after the pandemic, with 87.2 and 84.0%, respectively, indicating such preferences. As a result, the possibility of telework becoming a regular work style, beyond emergency measures, has become increasingly realistic. However, even during the peak of the pandemic, only 20–30% of teleworkers in Tokyo’s 23 wards worked entirely from home without commuting (Aoki et al., 2023a). The majority of workers adopted a hybrid model, combining both working from home and commuting to the office.

Since 2020, numerous of studies on telework as a workstyle that emerged in response to the COVID-19 pandemic have been conducted, and a range of review papers have summarized these findings (Beckel and Fisher, 2022; Crawford, 2022; Kniffin et al., 2021; Moglia et al., 2021; Mun et al., 2022; Vitória et al., 2022). These reviews highlight both the advantages and disadvantages of telework. While the overall impact of telework—whether positive or negative—depends on environmental and individual characteristics, it is clear that challenges related to communication and social isolation have arisen alongside its adoption. Nonetheless, many studies suggest that telework has the potential to improve job satisfaction and contribute to enhanced health and wellbeing. Additionally, following the pandemic, further research and policy implementation are needed to develop strategies that maximize the benefits of telework while mitigating its drawbacks. Mun et al. (2022) emphasize that telework became essential for organizations to maintain operations and ensure employee safety during the pandemic. Although telework was an option for some before COVID-19, the pandemic established it as a standard practice for many. Telework offers flexibility, granting employees greater control over their schedules, which can increase productivity and autonomy. However, challenges arose, particularly for those unaccustomed to remote work, such as difficulties in maintaining work-home boundaries and insufficient access to necessary physical equipment. Kniffin et al. (2021) identified several emergent changes associated with telework, including reduced travel costs and commute times, improved work-life balance for some employees, challenges in communication and collaboration in virtual settings, and blurred boundaries between work and personal life. They emphasized the need for long-term research on the impact of these changes on job productivity, creativity, and employee wellbeing. Moglia et al. (2021) discussed both the advantages and disadvantages of telework. Advantages include its potential to mitigate climate change, reduce traffic congestion, improve work-life balance, offer flexible work arrangements, enhance community resilience, and allow women and individuals in remote areas to maintain employment. However, disadvantages such as increased household energy consumption, environmental rebound effects, extended sedentary time, overwork, stress, social isolation, work–family conflict, and deterioration of labor rights were also noted. They proposed, based on their review and SWOT analysis, that addressing these challenges while capitalizing on telework’s benefits is essential for advancing toward the achievement of the SDGs. Beckel and Fisher (2022) state that telework offers employees flexibility, enabling them to manage their work environment, which can improve work-life balance, reduce commuting stress, and minimize exposure to office politics. Additionally, telework can provide health benefits such as reduced stress and lower blood pressure. However, they also warn that telework can blur the boundaries between work and personal life, potentially leading to work–family conflicts, social isolation, and diminished social support as employees feel less connected to their colleagues. Vitória et al. (2022) also found that telework brings significant changes, providing flexibility and improving job satisfaction, but also exacerbating work–family conflicts due to blurred boundaries and social isolation. They suggest several strategies for managing these challenges and maintaining a healthy balance between work and home life. Crawford (2022) indicated that while the precise impacts of telework on employee wellbeing remain unclear, telework appears to have a generally positive effect on short-term wellbeing and offers opportunities for more flexible and proactive work design. He concludes that further research should specifically focus on work design approaches that enhance both wellbeing and productivity, while also considering the environmental sustainability implications of reduced office work and the shift to working from home.

### 2.2 Telework and its impact on time use and work-life balance

Teleworking reduces time spent on commuting and grooming, enabling more flexible time use allocation, especially for leisure and household activities (Pabilonia and Vernon, 2020; Restrepo and Zeballos, 2020; Stiles and Smart, 2021). Restrepo and Zeballos (2020) identified differences in time use among U.S. teleworkers, who spent less time on work and more on leisure, sleep, and food-related activities. Teleworkers also reported increased time spent with family (Pabilonia and Vernon, 2020). Engaging in leisure has been shown to enhance mood, increase interest, lower stress, and reduce heart rate, which collectively benefit overall health and subjective wellbeing in daily life (Brajša-Žganec et al., 2011; Zawadzki et al., 2015). During the COVID-19 pandemic in Sweden, Hallman et al. (2021) observed that those working from home experienced longer sleep durations compared to office-based work, which may positively impact health. Additionally, flexible work arrangements contribute to stress reduction and higher job satisfaction, highlighting the importance of flexibility in personal and professional domains (Ray and Pana-Cryan, 2021).

Telework presents both opportunities and risks in relation to work-life balance and potential conflicts (OECD, 2013). For instance, telework has been linked to greater life satisfaction, with improvements in work-life balance acting as a mediating factor (Tan et al., 2024). During the pandemic, telework was frequently cited for its positive effect on work-life balance (Ipsen et al., 2021). While telework appeared to enhance wellbeing, imbalances in work-life boundaries also posed risks to wellbeing (Parent-Lamarche and Boulet, 2021). Conversely, telework’s impact on personal life includes challenges such as blurred boundaries between work and private life, mutual encroachment, increased household responsibilities, and imbalanced burdens or tensions within families (Nemțeanu and Dabija, 2023; Palumbo, 2020; Vitória et al., 2022). Zakhem et al., 2022 reported that COVID-19 blurred work-family role boundaries, negatively impacting performance and wellbeing; however, job autonomy helped to reduce work–family conflict by giving individuals greater control over their tasks, thereby enhancing both performance and wellbeing. Metselaar et al. (2023) also indicated that telework can lead to improved performance via autonomy and work-life balance satisfaction, especially when employees work from home.

### 2.3 Telework and its impact on communication

Telework has considerably impacted organizational communication, altering both formal and informal interactions among employees. This shift has affected collaboration dynamics, knowledge sharing, and interpersonal relationships, all of which are essential for effective teamwork (Mendonça et al., 2022; Yang et al., 2022). The perception of communication varies by demographics, occupation and the amount of time spent teleworking (Raišienė et al., 2020). While telework can reduce formal communication and real-time interactions, as well as connections between different teams (Yang et al., 2022), it also facilitates communication across geographically dispersed locations by leveraging ICT to overcome spatial and temporal barriers (Metselaar et al., 2023). However, telework often results in decreased communication with co-workers, potentially hindering knowledge sharing due to the spatial and temporal separation, and can contribute to feelings of social isolation (van der Meulen et al., 2019). Nakrošienė et al. (2019) found that reduced communication with colleagues correlated with higher productivity, suggesting that fewer interactions may benefit some. However, the impact of limited communication remains complex, and it is uncertain whether this reduction ultimately supports or hinders long-term workplace effectiveness. Telework may also lead to communication overload, perceptions of imagined surveillance, and increased work monitoring, all of which can affect mental health negatively (Mendonça et al., 2022). Furthermore, telework restricts informal communication opportunities, as employees miss out on the incidental interactions that occur naturally in physical office settings. These informal discussions now require intentional planning, which can reduce spontaneity and hinder relationship-building among colleagues (Viererbl et al., 2022; Watanabe et al., 2024). Effective management of telework requires addressing these communication challenges to ensure positive outcomes for both employees and organizations.

### 2.4 Telework and environmental impacts

Telework’s impact on CO_2_ emissions includes direct reductions in CO_2_ through decreased transportation demand, as well as trade-offs and rebound effects. The trade-off refers to CO_2_ emissions that arise from teleworking, which would not have occurred during regular office work. Examples include energy consumption from information and communication technologies (ICT) needed for telework, as well as increased energy use for heating, cooling, and lighting at home. The rebound effect, on the other hand, refers to the increase in environmental burden due to new behaviors, such as using the additional free time for non-work-related outings, moving to a larger suburban home and commuting longer distances by car, or engaging in energy-intensive leisure activities. Prior to the COVID-19 pandemic, multiple research groups conducted reviews on the effectiveness of telework as a CO_2_ reduction measure (Hook et al., 2020; Horner et al., 2016; O’Brien and Yazdani Aliabadi, 2020). These reviews indicated that, while the majority of existing studies show telework to be environmentally beneficial, there are cases where it has the opposite effect due to the presence of trade-offs and rebound effects. In Japan, a Nationwide survey revealed that teleworkers, compared to non-teleworkers, showed a marked increase in outings for purposes other than grocery or daily necessities shopping, particularly for leisure activities, with these outings occurring more frequently than before the pandemic (Ministry of Land, Infrastructure, Transport and Tourism of Japan, 2022b). Additionally, regarding the environmental impact over time, Kanamori et al. (2023) conducted a quantitative evaluation of CO_2_ emissions from work-from-home in urban areas of Japan and found that reducing environmental impact requires appropriate reductions in office space and home energy consumption. Furthermore, an increase in leisure time can have negligible effects on emissions, depending on the type of activity. Aoki et al. (2023b) pointed out the significance of the impact of telework-related video conferencing and highlighted the need to consider the increase in ICT-related emissions from video conferencing. As such, the environmental impact of telework is highly dependent on individual work and lifestyle patterns. Understanding changes in time use due to telework is crucial not only for assessing its social impacts but also for evaluating its environmental effects.

### 2.5 Conceptual framework

This study aims to explore the impact of teleworking on workers’ daily life and work patterns, particularly focusing on how telework affects time allocation, and intentions to continue teleworking. The Job Demands–Resources (JD-R) theory (Bakker and Demerouti, 2017, 2007), a widely used framework for understanding employee wellbeing and work behaviors, provides a relevant theoretical basis for this investigation. The JD-R theory posits that work demands, such as workload and complexity, act as stressors, while resources, including autonomy and organizational support, serve to mitigate these demands and enhance motivation. When resources are sufficient to meet demands, employees are more likely to experience improved productivity and wellbeing. Conversely, insufficient resources can exacerbate the negative impact of demands, leading to reduced performance and increased strain. Applying this theory to telework, this study examines the interplay between telework-related demands, such as task difficulty and workload, and resources like autonomy and flexible support systems, to understand their combined influence on productivity and wellbeing.

**Figure 1:**
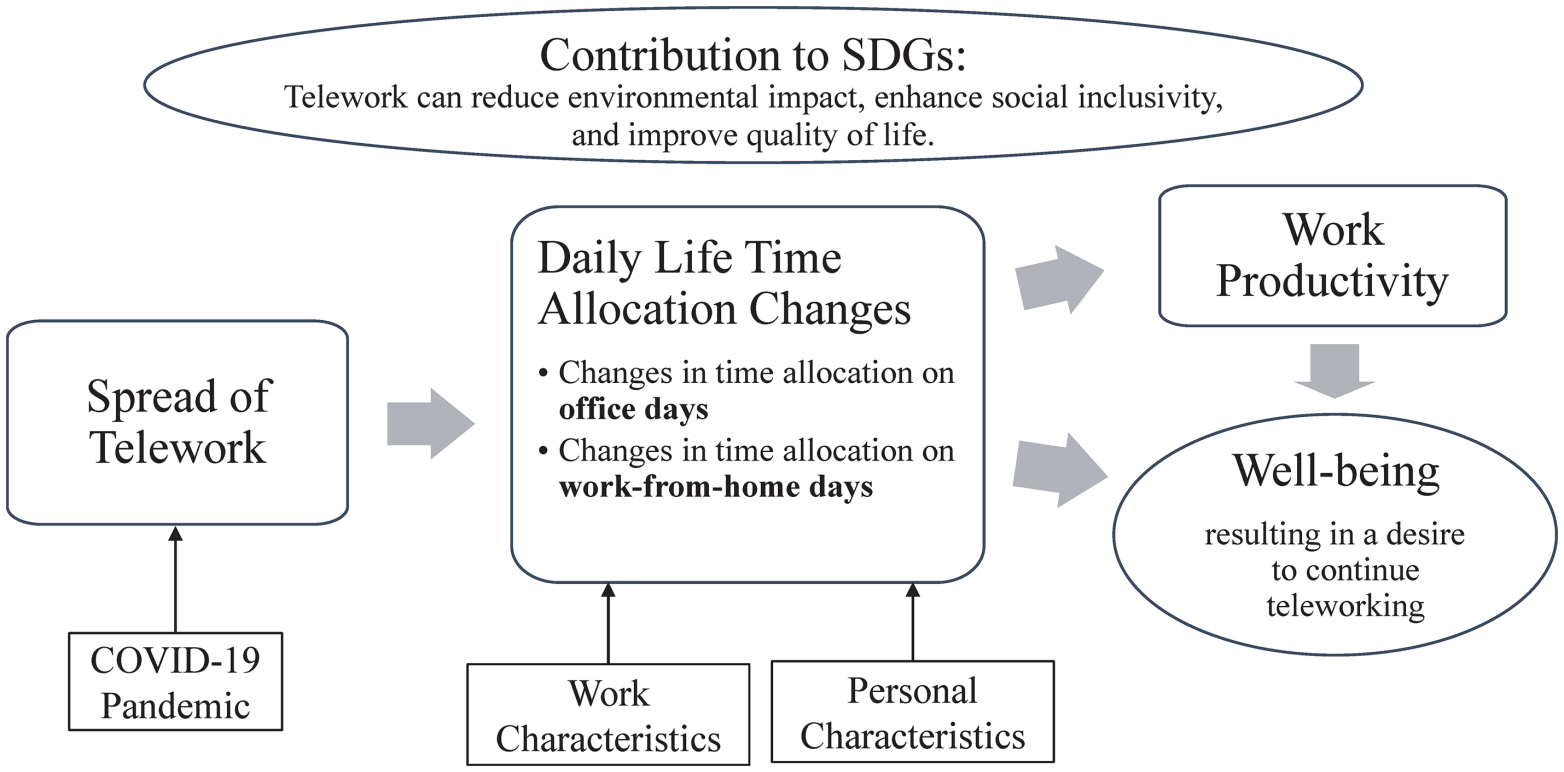
Conceptual framework of this study.

Figure 1 presents the conceptual framework of this study. The framework guides the analysis, outlining the influential factors and how telework affects work productivity and wellbeing. While the framework suggests potential relationships and dynamics, the precise nature of these relationships remains uncertain. To address this gap, the study focuses on several key questions to uncover the underlying mechanisms and broader implications of telework:

- Impact of teleworking on daily life time: How has the introduction of teleworking influenced changes in daily life, such as increasing leisure and rest time for workers? This question seeks to understand the broader implications of teleworking on personal time management and lifestyle changes.
- Variability in time allocation beyond telework days: What are the changes in time allocation that occur beyond the days designated for teleworking? For instance, does teleworking lead to increased workloads on office days to compensate for the flexibility of WFH days? This question examines the potential shifts in work patterns that are not immediately visible when focusing solely on telework days.
- Influence of work and personal characteristics: How do the nature of work and individual personality traits (especially those related to communication with others) influence the changes and effects experienced under teleworking? This question aims to identify the factors that moderate the impact of teleworking, suggesting that teleworking is not a one-size-fits-all solution.
- Work productivity and wellbeing: To what extent do the autonomy and flexibility afforded by telework, along with the intensity of telework implementation, enhance work productivity, and can these factors also promote individual wellbeing? This question seeks to explore whether varying levels of telework engagement influence productivity improvements and whether these benefits translate into broader personal wellbeing, contributing to a more sustainable and satisfying work style.
- Relationship between teleworking, personal time, and continuation preferences: How does the increase in leisure and rest time associated with teleworking, which may enhance wellbeing, influence workers’ preferences for continuing to telework? This question examines whether the additional personal time and the resulting improvement in wellbeing motivate workers to choose teleworking as a long-term work arrangement.

By addressing these questions, this study aims to provide a comprehensive understanding of the effects of teleworking on workers’ lives, contributing to the development of telework policies that enhance both work productivity and individual wellbeing while accommodating diverse work and personality needs.

## 3 Materials and methods

### 3.1 Questionnaire survey: details and design

In this survey, we focused on WFH as a form of telework. In 2020, when telework was most common, WFH accounted for the majority of telework and had the greatest impact on people’s lifestyles, therefore our focus was limited to WFH. In the questionnaire, we defined WFH as “working at home using a telecommunications network.” The survey was conducted in November 2021, targeting those who regularly engaged in WFH during 2021, a period when society had somewhat stabilized following the emergency adoption of telework due to the COVID-19 pandemic. To ensure the WFH frequency (number of days per week) reported by respondents closely reflected reality, we asked them about their most consistent period of WFH in 2021 at the beginning of the survey. Using frequency data from November 2020, which we had previously investigated (Aoki et al., 2023a), we allocated the number of respondents across three categories (1 day per week, 2–3 days per week, 4 or more days per week). We designed and developed the questionnaire and commissioned a research company to conduct the online survey. The survey was conducted entirely online, without any direct intervention or intrusion. Consequently, we obtained the respondents’ consent online, following the prescribed criteria set forth by the university.

### 3.2 Questionnaire survey: sample selection

To enable respondents to compare their experiences with the period before the COVID-19 pandemic, we targeted only those who had been working full-time at the same workplace from 2019 to the time of the survey. The survey targeted individuals in their 20–60s living in the Tokyo Metropolitan Region (Tokyo, Saitama, Chiba, Kanagawa, Ibaraki, Tochigi and Gunma prefectures), which is centered on Tokyo and is located in the surrounding area of Japan’s capital. Participants were selected through screening to ensure they met these criteria. We conducted the survey online, a method widely used in Japan for its ability to reach larger and more diverse populations (Kurisu and Bortoleto, 2011). In this approach, survey invitations are sent to a targeted group of registered respondents meeting the study’s criteria via a survey company, with participants earning points as incentives for completing the questionnaire. It is important to note that the sample is not representative of the Tokyo Metropolitan Region as a whole but is confined to a specific subset of participants who fulfilled the predefined study criteria.

### 3.3 Contents of the questionnaire survey

The questionnaire consisted of five parts: (1) the working situation of telework, including the frequency of WFH before the COVID-19 pandemic and at the time of the survey; (2) the working situation on WFH and office days during the WFH period, covering aspects such as autonomy, workload, productivity, and working hours; (3) the daily life situation on WFH and office days during the WFH period, detailing changes in daily life time (e.g., housework, leisure time); (4) personal attributes, including age, gender, living situation, occupation, and personality measured using the Japanese version of the Ten Item Personality Inventory (TIPI-J; Gosling et al., 2003; Oshio et al., 2012). The items in sections (2) and (3), focusing on participants’ working and living styles, were evaluated on a five-point scale: “increased,” “slightly increased,” “no change,” “slightly decreased,” and “decreased.” For item (3), respondents were also asked whether they had previously engaged in the activity with response options including: “I did not do it before and do not do it now,” “I started doing it,” “it increased,” “no change,” and “it decreased.”

In this survey, it was essential to consider the differences between an individual’s WFH days and office days. Therefore, we used a relative evaluation based on respondents’ subjective assessments, rather than employing a method like the diary approach, which records behavior as an absolute value on a specific day. This approach allowed us to capture perceived changes due to WFH as typical changes over the survey period, reducing respondent burden and minimizing survey dropouts. While capturing individual productivity (especially for clerical or intellectual tasks) using relative changes is challenging, it is possible to understand these changes by asking respondents to make a subjective comparison, similar to the approach taken by Morikawa (2022).

### 3.4 Statistical analysis

Data analysis was performed using IBM SPSS Statistics ver.28, focusing on principal component and cluster analyses to identify patterns in daily time allocation and work styles. In the principal component analysis, we inputted the variables that asked about the way of working and daily life time on WFH days and at office days during the WFH period, and showed the changes by aggregating them according to their characteristics. As a method, we used the Varimax rotation, which rotates the data in a way that is suitable for the data while maintaining the orthogonality of the axes for aggregating the information. Next, cluster analysis (Ward’s method) was conducted to characterize how the respondents changed in terms of the characteristics of their work style and daily life time obtained through principal component analysis, and to categorize them into groups that showed similar changes. By analyzing the characteristics of these groups, we clarified what kind of changes were occurring in people with what kind of characteristics due to WFH. We also employed statistical methods, including chi-square tests and ANOVA, to examine variations across groups, allowing us to identify distinct change patterns among respondent groups.

## 4 Results

### 4.1 Work and life style changes in WFH

We received 1,500 valid responses. Regarding the basic attributes of the respondents, 1,191 were male, and 309 were female, with an average age of 50.9 years (SD = 9.42), the most common age group being those in their 50s. In terms of living situations, 377 respondents lived alone, 400 lived with one other person, 365 lived with two others, and 358 lived with three or more people. As for housing, 602 respondents lived in detached houses, while 898 lived in apartment complexes. About 33% lived with children, and 27% owned pets. Regarding occupations, the largest number of respondents were regular employees (686 respondents), followed by 397 in managerial positions, 87 company executives, 103 self-employed individuals, 90 public servants or educational/institutional workers, 100 temporary or contract employees, and 37 in other categories.

The frequency of telework in 2021 was as follows: 307 respondents worked from home an average of one day per week, 345 for two days, 321 for three days, 166 for four days, and 361 for five or more days (based on the survey design). Furthermore, 16% (240 respondents) had already been teleworking regularly since 2019, prior to the pandemic, while 9% (137 respondents) only began teleworking regularly in 2021, having not done so during the height of the pandemic in 2020.

#### 4.1.1 Telework experience and changes in work styles

**Figure 2:**
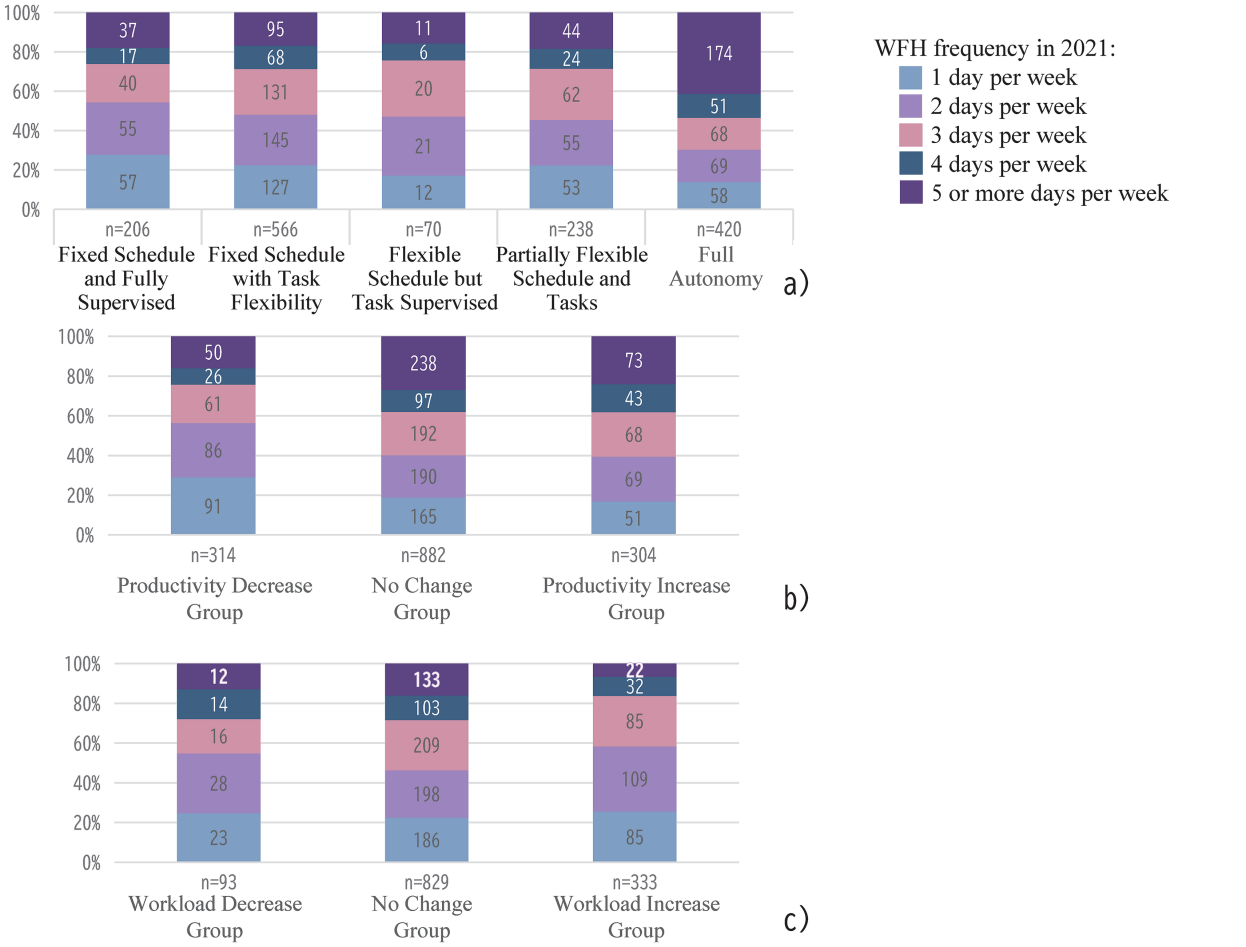
Distribution of Work-from-Home Days by Work Characteristics. a) Autonomy in Telework. b) Work productivity in Telework. c) Workload on Office Days.

We investigated respondents’ autonomy in managing work and personal time while teleworking. Specifically, we asked whether they could decide their own working hours and whether their activities were supervised (i.e., whether personal activities during working hours were completely prohibited and supervised or if they were allowed some flexibility in what they wanted to get done). The question offered five response options, with the highest level of autonomy being the ability to decide entirely at one’s discretion. As shown in Figure 2A, the most common response was that working hours were determined by the employer (*n* = 566), but they were allowed some flexibility in what they wanted to get done. A significant correlation was found between the number of telework days and the level of autonomy over time and tasks (*χ*^2^ = 115.39, *df* = 16, *p* = 0.000, *V* = 0.14). Those who teleworked five or more days per week reported the highest levels of autonomy, while those who teleworked only one day per week reported the highest levels of supervision. We also compared respondents’ perceptions of their workload, work productivity, and working hours (excluding commuting time) during the telework period with those before the pandemic, both on WFH and office days. Across all categories, more than half of the respondents reported no change, regardless of telework frequency. However, there was significant variation in productivity (*χ*^2^ = 35.28, *df* = 8, *p* = 0.000, *V* = 0.11). Those who teleworked one day per week or less were more likely to report a decrease in productivity, while those teleworking four or more days per week were more likely to report an increase (see Figure 2B). Furthermore, a significant correlation was found regarding increased workloads on office days during the telework period (*χ*^2^ = 29.10, *df* = 8, *p* = 0.000, *V* = 0.11). Although more than half of the respondents reported no change, those who teleworked three days or fewer were more likely to report an increase in workload (see Figure 2C).

Table 1 presents the results of a six-point Likert scale that measured 10 items related to work attitudes during telework, ranging from “completely disagree (1)” to “completely agree (6).” Respondents were categorized into three groups based on their productivity responses: the “No Change Group,” the “Productivity Increase Group, “and the “Productivity Decrease Group.” Multiple comparisons (Tukey’s HSD) between the Productivity Increase Group and Decrease Group revealed significant differences in seven items, which included both positive aspects and challenges. The Productivity Decrease Group reported greater difficulties with switching between work and personal life and with communication, in addition to stating that their job duties were inherently unsuitable for telework. These findings align with prior reports showing that individuals with pre-pandemic telework experience tended to exhibit increased productivity, while those forced into telework during the COVID-19 pandemic struggled with lower efficiency (Morikawa, 2022; Nemțeanu and Dabija, 2023).

**Table 1:** Recognition of WFH work style and differences in perceived productivity changes while WFH.

	No change group	Productivity increase group	Productivity decrease group	Multiple comparisons (Tukey HSD)
	Mean	Mean	Mean	*p*
Able to concentrate on work	4.1	**4.0**	**3.8**	**0.030**
No problems with work environment	4.0	**3.8**	**3.3**	**0.000**
Autonomy is desirable	4.3	4.2	4.3	0.240
Requires additional work or effort	3.2	**3.4**	**3.7**	**0.000**
Privacy concerns	3.0	3.0	3.0	0.880
Must pay for expenses personally	3.4	3.7	3.6	0.360
Work content is unsuitable	2.8	**2.8**	**3.4**	**0.000**
Difficult to switch between work and personal time	3.5	**3.5**	**4.1**	**0.000**
Decreased informal communication with others	3.8	**3.9**	**4.3**	**0.000**
Difficulty in communicating about work	3.6	**3.6**	**4.1**	**0.000**

Tukey’s HSD test, Tukey’s honestly significant difference test. Analysis results between the productivity increase group and the productivity decrease group. Bold values indicate statistically significant results (*p* < 0.050).

Although the survey period spanned more than a year after the initial state of emergency declaration, allowing some degree of preparation, the data suggest that a fully optimized telework environment had not yet been established. Individuals who had prior experience with telework or whose job duties were entirely compatible with remote work appeared to have adapted more efficiently. Additionally, the finding that workloads and working hours tended to increase on office days during the telework period is notable. This may suggest that tasks perceived as executable only in the office are hindering the flexibility of telework, or it could indicate that administrative tasks and communication-intensive work are being concentrated on office days to maximize efficiency. The impact of telework on productivity and job satisfaction may depend on the efficient division of tasks, particularly those requiring interaction with others. Without restructuring these tasks, the decrease in productivity during telework may persist. Therefore, supporting the effective spread of telework will require not only improvements in telework technology and management but also a comprehensive restructuring of task management, categorization, and evaluation, focusing on the broader scope of job responsibilities.

#### 4.1.2 Telework experience and changes in lifestyle

**Figure 3:**
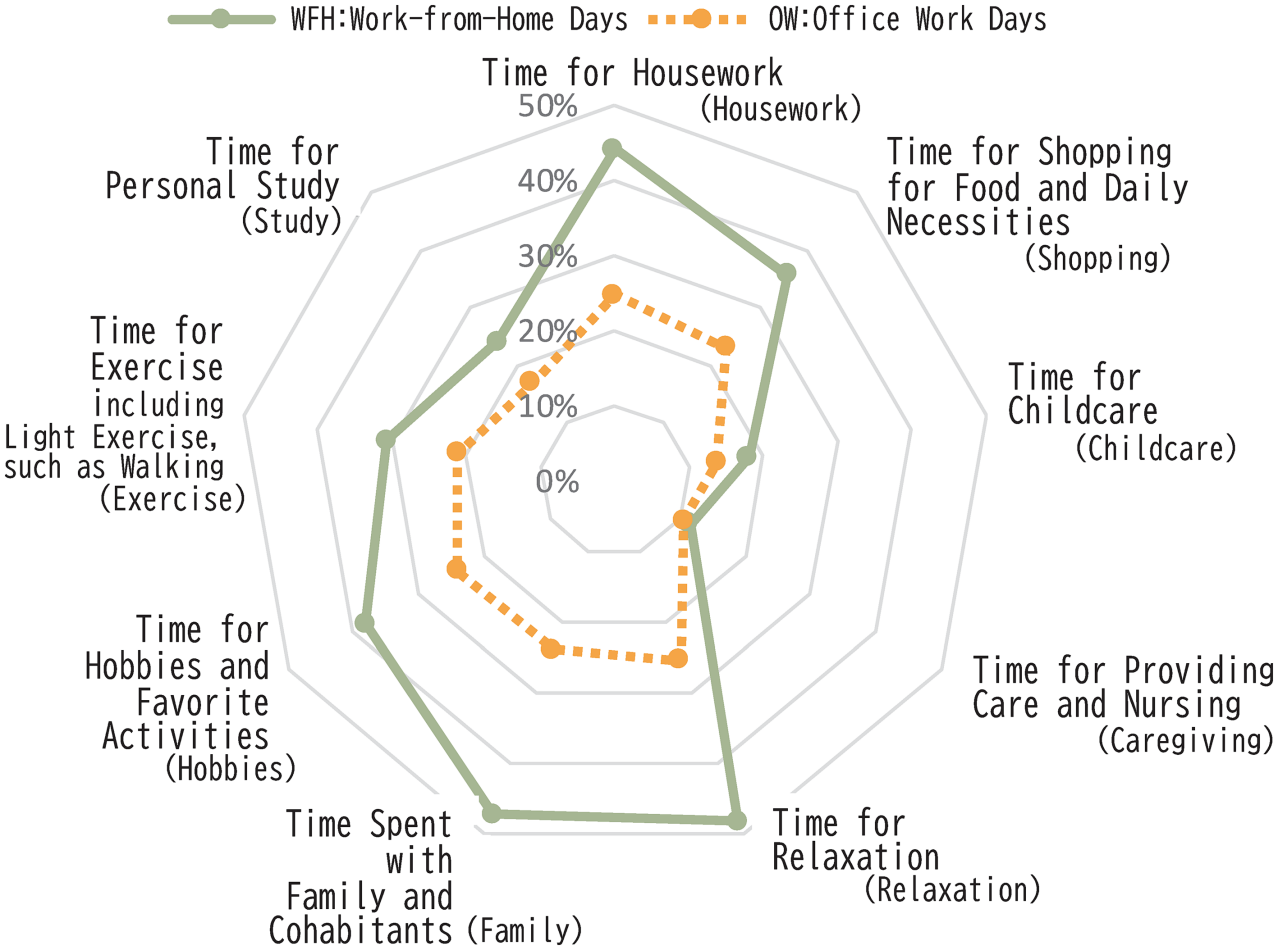
Proportion of respondents reporting increases in nine categories of daily life time during the telework period.

We analyzed changes in nine categories of daily life, including housework and leisure, for both WFH and office days. In all categories, “No change” was the most frequent response. Except for “relaxation time” on WFH days (47.3%), over half of the respondents reported no change across the categories (50.6–87.2% on WFH days, 55.7–73.1% on office days). However, we also observed an increase in time spent across all categories, even on office days during the telework period. To assess these changes, we asked respondents about changes in time allocation for each category using the phrasing “time spent on [activity].” Figure 3 shows the percentage of respondents who reported an increase in time spent on each activity. Notably, a significant number of respondents reported an increase in two rest-related items on WFH days: time for relaxation and time spent with family or cohabitants.

**Figure 4:**
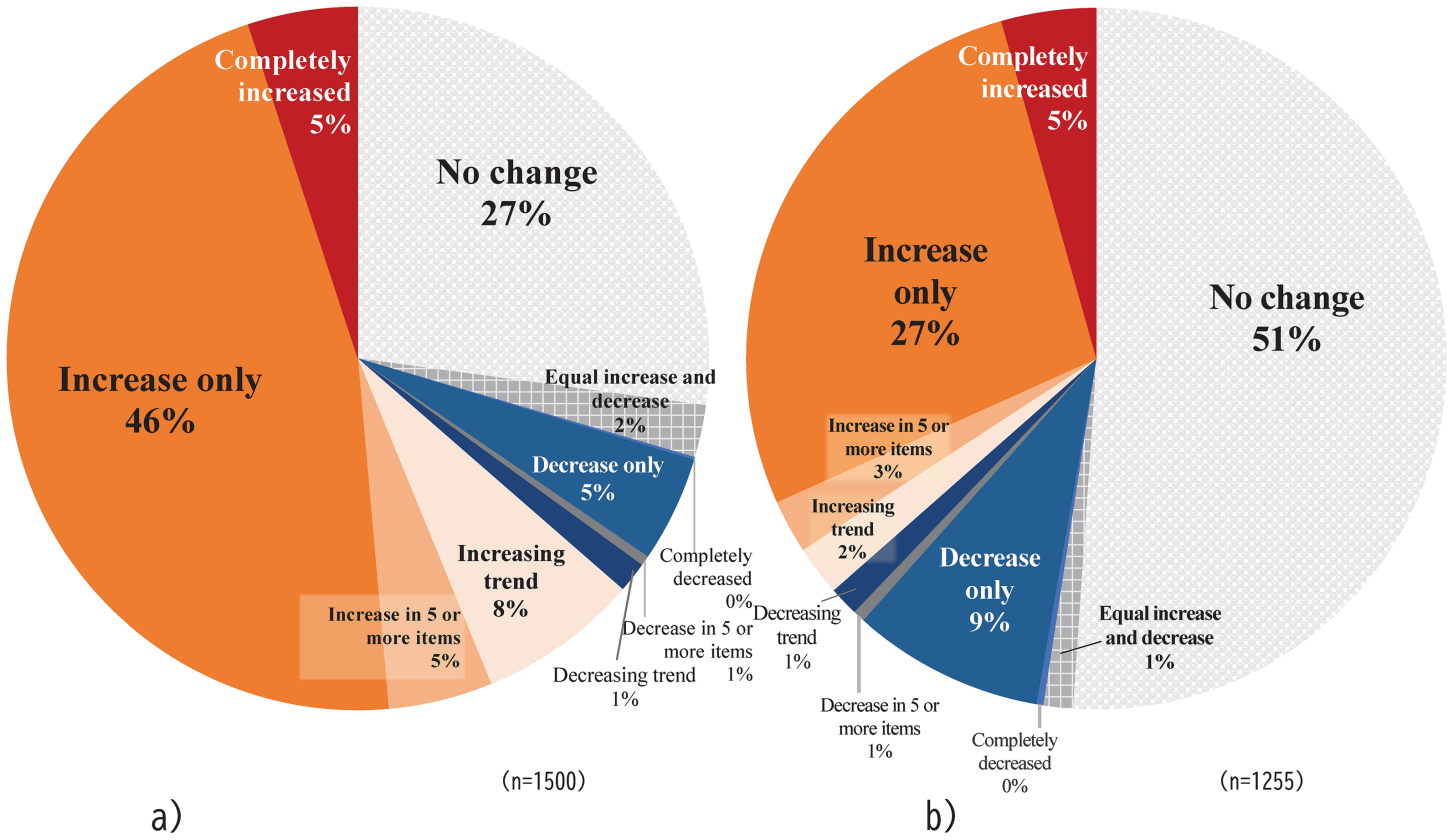
Proportion of Changes in Nine Categories of Daily Life Time During the Telework Period. a) WFH: Work-from-Home Days. b) OW: Office Work Days.

Figure 4 classifies respondents into 10 categories based on the ratio of increase and decrease across the nine categories, as well as the overall balance of changes in time allocation (e.g., no change, equal increase and decrease, or a stronger tendency toward either increase or decrease). On WFH days, more respondents reported an increase in daily life time. When combining those who reported an increase across all nine categories (“Complete increased”) and those who reported no decreases (“Increase only”), the majority (51%) experienced an overall increase in time allocation. Including respondents who had some decrease but more increase, 64% of respondents experienced a net increase in time spent. On office days, while the majority reported no change, more respondents reported an increase in time spent rather than a decrease. However, the number of respondents reporting a decrease in time spent was higher on office days compared to WFH days. Few respondents reported both increases and decreases across different time categories. Most individuals fell into groups that reported either “no change” across all categories, “increase only or no change, “or “decrease only or no change” in their overall daily time allocation. These results indicated that the introduction of telework has led to distinct changes in many workers’ daily time allocation, including an increase in leisure and rest time.

Among the nine categories, activities like childcare and caregiving might not seem relevant to all respondents, as some individuals may not have cohabitants with care needs or direct caregiving responsibilities. However, caregiving activities in the survey were defined broadly, including support for relatives living in separate households or responsibilities shared with other family members. This approach ensured that even those who might not initially consider themselves as caregivers were included in the analysis. Regardless of individual attributes or circumstances, all respondents were asked about these categories, and their responses were included in the subsequent analysis, just as with the other activity categories.

### 4.2 Characteristics of WFH workers

#### 4.2.1 Aggregation of categories for changes in lifestyle and work patterns

To analyze relative changes compared to respondents’ past experiences, the survey questions (2) and (3), which inquired about changes in lifestyle and working hours, were converted from five response options to three levels of variables. Responses for question (2) such as “increased” and “slightly increased, “and for question (3) “started doing” and “increased, “were categorized as “increase.” Responses such as “slightly decreased” and “decreased” from question (2), and “decreased” from question (3), were categorized as “decrease.” Finally, responses like “no change” from question (2) and “have not done before or now” and “no change” from question (3) were categorized as “no change.” Using the data from 26 items related to changes in lifestyle and work, we conducted a principal component analysis on 1,255 respondents who had office days during the telework period. Upon examining the results, the item for “sleep time on office days” had low communality, so it was excluded, and a reanalysis was conducted using the remaining 25 items. Looking at the response distribution, 981 of the 1,255 respondents indicated no change in sleep time, showing the greatest differences toward “no change” across all items. The analysis results, summarized into a six-factor structure based on changes in eigenvalues and analytical feasibility, are shown in Table 2. In the table, the items are labeled with “OW: office work” or “WFH” at the beginning to indicate whether they refer to office days or WFH days, followed by an abbreviation of the lifestyle or work item. The factors separated into items related to lifestyle and work on WFH days and office days, leading to the following names for the factors: Factor I: Lifestyle on Office Days, Factor II: Lifestyle on WFH Days, Factor IV: Work on Office Days, and Factor V: Work on WFH Days. Additionally, factors that were grouped based on the content of the items rather than the presence or absence of office work were named Factor III: Family Care, and Factor VI: Self-Actualization Time. Based on this principal component analysis, we calculated the factor scores for each respondent’s changes in work and lifestyle across these six factors.

**Table 2:** Results of principal component analysis on work and life time changes between WFH and office work days.

	I	II	III	IV	V	VI
OW relaxation time	**0.80**	0.18	−0.08	0.05	0.00	0.18
OW family time	**0.78**	0.20	0.13	0.00	−0.03	−0.04
OW hobbies time	**0.77**	0.12	−0.05	0.00	−0.06	0.33
OW housework time	**0.75**	0.17	0.29	0.01	−0.03	−0.07
OW shopping time	**0.74**	0.15	0.32	−0.02	−0.07	−0.04
OW study time	**0.67**	0.00	0.19	−0.01	−0.05	0.39
OW exercise time	**0.55**	−0.02	0.14	−0.01	−0.07	0.47
WFH relaxation time	0.16	**0.72**	−0.07	0.03	0.12	0.30
WFH family time	0.10	**0.68**	0.23	0.02	0.01	0.02
WFH housework time	0.10	**0.61**	0.40	−0.10	−0.03	0.00
WFH sleep time	−0.06	**−0.58**	0.17	0.15	0.07	−0.11
WFH hobbies time	0.19	**0.56**	0.00	0.01	0.02	**0.56**
WFH shopping time	0.17	**0.56**	0.42	0.00	−0.05	0.01
WFH childcare time	0.09	0.24	**0.75**	−0.03	−0.05	0.13
WFH caregiving time	0.14	0.08	**0.71**	−0.06	−0.06	0.23
OW childcare time	**0.54**	−0.04	**0.57**	−0.03	−0.03	0.11
OW caregiving time	0.44	−0.07	**0.56**	−0.07	0.03	0.20
OW work amount	0.01	−0.04	−0.03	**0.86**	0.05	−0.01
OW productivity	0.00	−0.07	−0.03	**0.85**	0.08	−0.02
OW work time	0.01	−0.03	−0.06	**0.82**	0.16	0.01
WFH work amount	−0.05	−0.01	−0.05	0.03	**0.89**	0.01
WFH productivity	−0.07	−0.03	0.01	0.06	**0.81**	−0.14
WFH work time	−0.03	0.04	−0.07	0.22	**0.80**	0.07
WFH exercise time	0.12	0.11	0.25	−0.01	−0.02	**0.72**
WFH study time	0.19	0.31	0.26	−0.01	−0.06	**0.61**
Factor contribution	4.39	2.65	2.53	2.22	2.16	1.98
Cumulative contribution (%)	17.60	28.20	38.30	47.20	55.80	63.70

WFH, Work-from-Home; OW, Office Work. Bold values indicate eigenvalues of 0.50 or higher.

#### 4.2.2 Grouping by changes in lifestyle and work patterns

A cluster analysis was conducted using the factor scores calculated for each respondent in the previous section. The dendrogram output was cut at specific vertical lines to identify points where branching occurred, and divisions changed based on the distance between items. Five clusters, each demonstrating distinct characteristics regarding work styles and lifestyles, were identified. Additionally, a separate group was created for those who worked entirely from home, who were excluded from the analysis in the previous section, resulting in a total of six groups for further analysis. Each group is named based on its unique characteristics in changes in daily life time and work conditions: “Housework and Rest Increase Group (G1),” “Unchanged Group (G2),” “Increase Only on WFH Days Group (G3),” “Overall Increase Group (G4),” “Unsuitable for WFH Group (G5),” and “Full WFH Group (G6).” Table 3 shows the percentage and total number of respondents in each group based on the frequency of WFH, representing the longest period of telework in 2021. There were significant differences in the frequency of WFH (*χ*^2^ = 361.1, *df* = 10, *p* = 0.000, *V* = 0.35), with 43% of respondents belonging to the “Unchanged Group,” followed by 16% in the “Full WFH Group” and 12% in the “Increase Only on WFH Days Group.” The proportion of respondents working from home one day per week was higher in the “Unchanged Group,” but this group also had a higher percentage of respondents working four or more days per week, second only to the “Full WFH Group.” This suggests the existence of two distinct subgroups: those whose lifestyle did not change because they rarely engaged in telework, and those whose lifestyle remained unchanged because they fully adapted to telework.

**Table 3:** Number of people in each WFH group and percentage of WFH frequency.

WFH groups	Percentage of each group for the frequency of WFH per week			Total *n* (% of respondents)
	Once a week	2–3 times a week	4 or more times a week	
HR increase group (G1)	13.8%	58.6%	27.6%	116 (7.7%)
Unchanged group (G2)	26.7%	45.3%	28.0%	651 (43.4%)
WFH increase only group (G3)	20.2%	56.1%	23.7%	173 (11.5%)
Overall increase group (G4)	22.6%	56.2%	21.2%	146 (9.7%)
Unsuitable WFH group (G5)	21.3%	60.9%	17.8%	169 (11.3%)
Full WFH group (G6)	5.3%	8.6%	86.1%	245 (16.3%)

WFH, Work-from-Home; HR, Housework and Rest.

**Figure 5:**
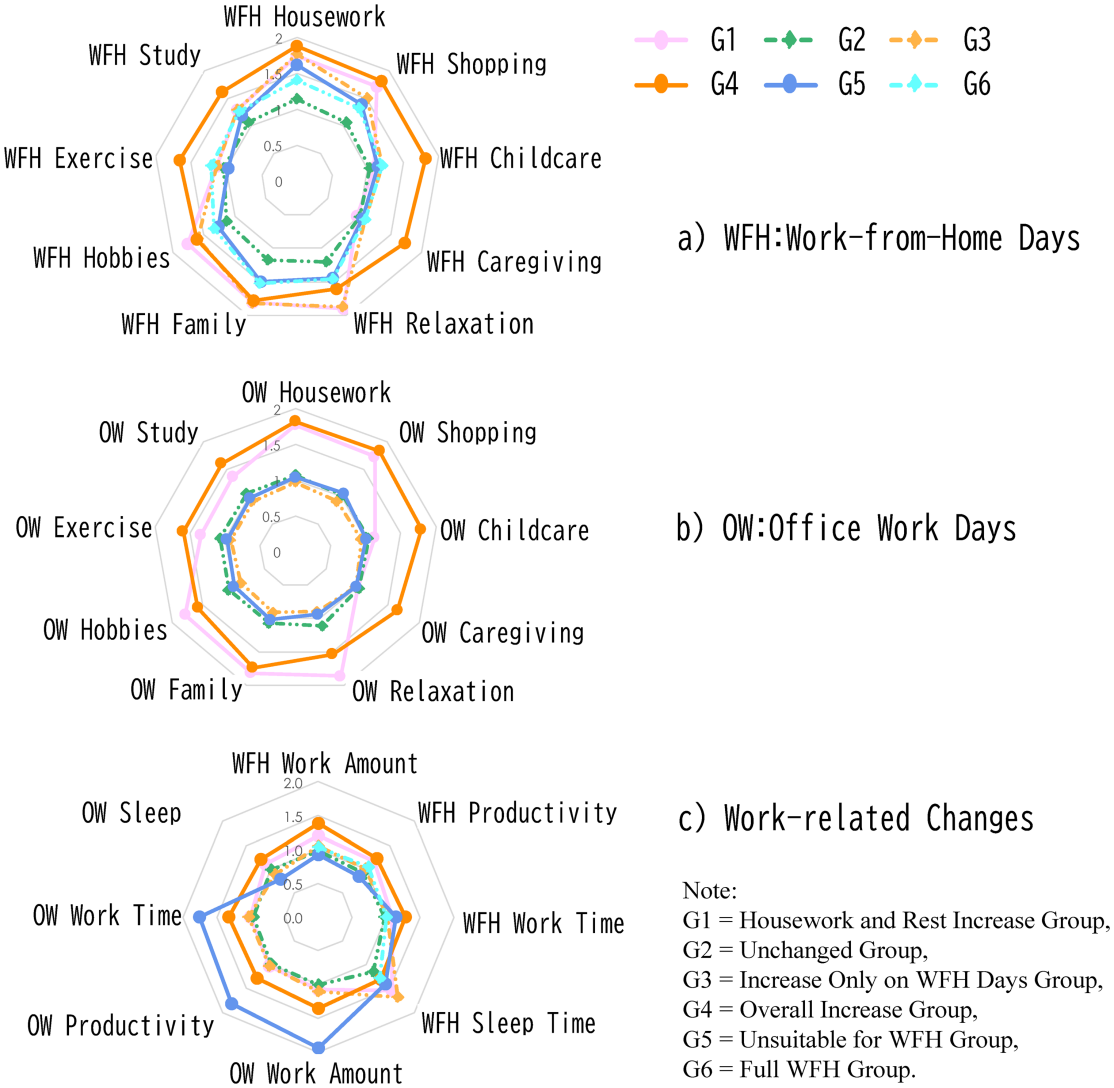
Overview of Itemized Changes in Daily Life Time and Work During the Telework Period. a) WFH: Work-from-Home Days. b) OW: Office Work Days. c) Work-related Changes.

Additionally, Figure 5 shows the mean values for each group, using variables where “decrease” was coded as 0, “no change” as 1, and “increase” as 2. In the chart, movement toward the outer edge indicates an increase, while movement toward the inner edge indicates a decrease. The “Overall Increase Group” showed an increasing trend across all items, while both the “Housework and Rest Increase Group” and the “Increase Only on WFH Days Group” exhibited a notable increase in relaxation time, time with family, and time for hobbies. Furthermore, except for the “Housework and Rest Increase Group” and the “Overall Increase Group,” all groups showed little change on office days, with values close to 1. Looking at the chart for work-related changes, the “Unsuitable for WFH Group” stood out. Compared to before, this group experienced a decrease in work hours, workload, and productivity on WFH days, while office days saw an increase in both work hours and workload, along with a decrease in sleep time. This indicates that this group struggled to adjust to telework, leading to an increased burden on office days. They also recognized that their productivity was higher on office days, reaffirming the challenges they faced with telework.

#### 4.2.3 Characteristics of telework-related change groups

The results of the chi-squared test between the classified groups and individual attributes are shown in Table 4. Significant differences were observed at the 5% level for gender, age group, number of cohabitants, housing type, presence of children, presence of pets, and occupation. However, the effect size (*V*) was around 0.1, indicating that these individual attributes had a small impact. Therefore, the classification of groups appears to be more closely related to job characteristics and individual perceptions rather than personal attributes. Nevertheless, certain characteristics can still be observed, and the following provides an excerpt of basic information about the individuals in each group.

**Table 4:** Personal attributes, continuation preferences, and chi-square test in WFH groups.

	*χ* ^2^	*df*	*p*	*V*
Gender	24.1	5	**0.000**	0.127
Age group	43.9	20	**0.002**	0.086
Number of cohabitants	50.2	20	**0.000**	0.092
House type (detached/apartment)	13.9	5	**0.016**	0.096
Presence of children	19.2	5	**0.002**	0.113
Presence of pets	22.2	5	**0.000**	0.122
Occupation	85.6	30	**0.000**	0.107
Frequency of continuation preference	235.4	15	**0.000**	0.229

*χ*^2^, chi-square test; df, degree of freedom; *V*, Cramér’s *V*. Bold values indicate statistically significant results (*p* < 0.050).

**Figure 6:**
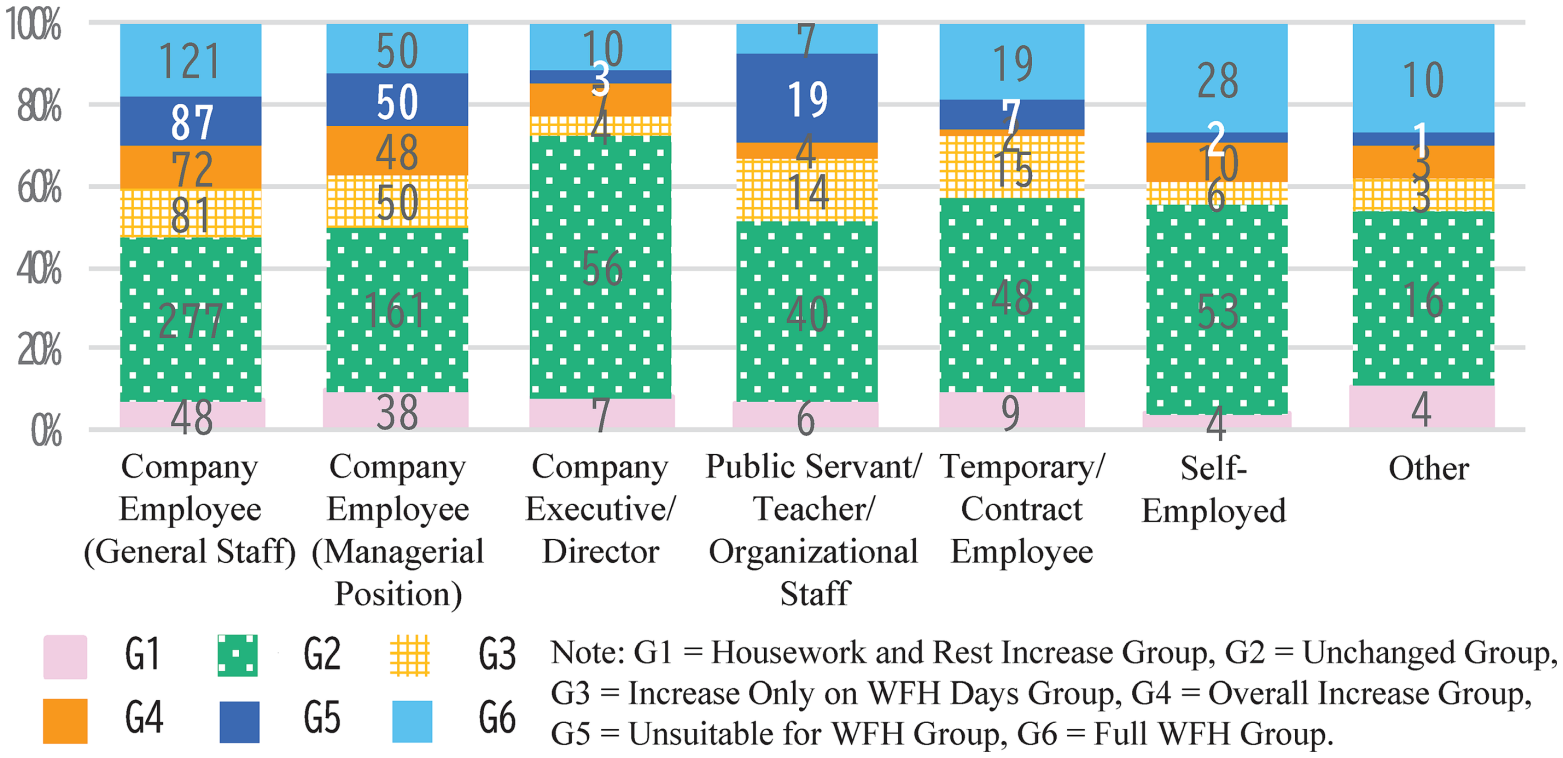
Distribution of occupations across WFH groups.

The “Overall Increase Group” is distinctive, with many respondents living in households with more cohabitants, in detached houses, in their 40s, and living with children or pets. This is typically a family consisting of parents and children in a detached house, where increased time at home likely led to more shared time with family members. Additionally, the “Unsuitable for WFH Group” has a higher proportion of women. This reflects survey results showing that many women in this group reported decreases in time spent shopping, exercising, and studying, both on WFH days and office days. This group also had a higher proportion of respondents living in apartment complexes, with a notable percentage living alone, second only to the “Unchanged Group.” In terms of age, younger individuals, especially those in their 40s or younger, were more prevalent in the “Unsuitable for WFH Group,” following the “Overall Increase Group.” Although company employees were the largest occupational group across all clusters, the proportion of public servants, teachers, and organizational staff was notably higher in the “Unsuitable for WFH Group” (Figure 6). In terms of occupation, the “Full WFH Group” had a higher proportion of self-employed individuals, while the “Unchanged Group” had a higher percentage of company executives and directors. This suggests that individuals in these two occupations were either well-suited for telework or were able to maintain high telework frequency without significant changes in their lifestyle.

Furthermore, personality traits were assessed using the Japanese version of the Ten-Item Personality Inventory (TIPI-J; Gosling et al., 2003; Oshio et al., 2012), which measures the Big Five personality traits. Each of the five subscales consisted of two items, and respondents were asked to answer using a seven-point scale ranging from “Disagree strongly” to “Agree strongly.” Scores for each dimension were calculated on a 2–14-point scale, and comparisons were made across the groups. The TIPI-J has been validated in Oshio et al. (2012), demonstrating correlations with existing Big Five personality measures and confirmed test–retest reliability, ensuring its validity and reliability. In this study, we strictly adhered to the methodology prescribed by the developers, including the use of the specified questionnaire items and scoring methods. We presented the information of the used scales and the results of the ANOVA in Table 5. These results revealed significant differences in agreeableness and conscientiousness. The “Overall Increase Group” had the lowest average score for agreeableness (8.89), while the “Unsuitable for WFH Group” had the highest (9.83). For conscientiousness, the “Overall Increase Group” also had the lowest average score (8.27), while the “Productivity Decrease Group” (and the “Unsuitable for WFH Group”) had the highest scores (8.84 and 8.80, respectively). These results suggest that individuals with higher levels of agreeableness might have faced challenges in adjusting to telework, possibly due to the unfamiliar social interactions required online. Similarly, individuals with high conscientiousness may have struggled to balance work and personal time, being overly diligent, which prevented telework from effectively improving their work or lifestyle.

**Table 5:** Big five scale information and results of one-way ANOVA in WFH groups.

	Mean	SD	*F*	*df*	*p*	Partial *η*^2^
Extraversion	7.75	2.34	1.32	5, 1,494	0.255	0.004
Agreeableness	9.32	2.03	6.51	5, 1,494	**0.000**	0.021
Conscientiousness	8.53	2.09	3.21	5, 1,494	**0.007**	0.011
Neuroticism	7.42	2.09	2.15	5, 1,494	0.057	0.007
Openness	7.99	2.12	0.48	5, 1,494	0.790	0.002

df, degrees of freedom. Bold values indicate statistically significant results (*p* < 0.050).

**Figure 7:**
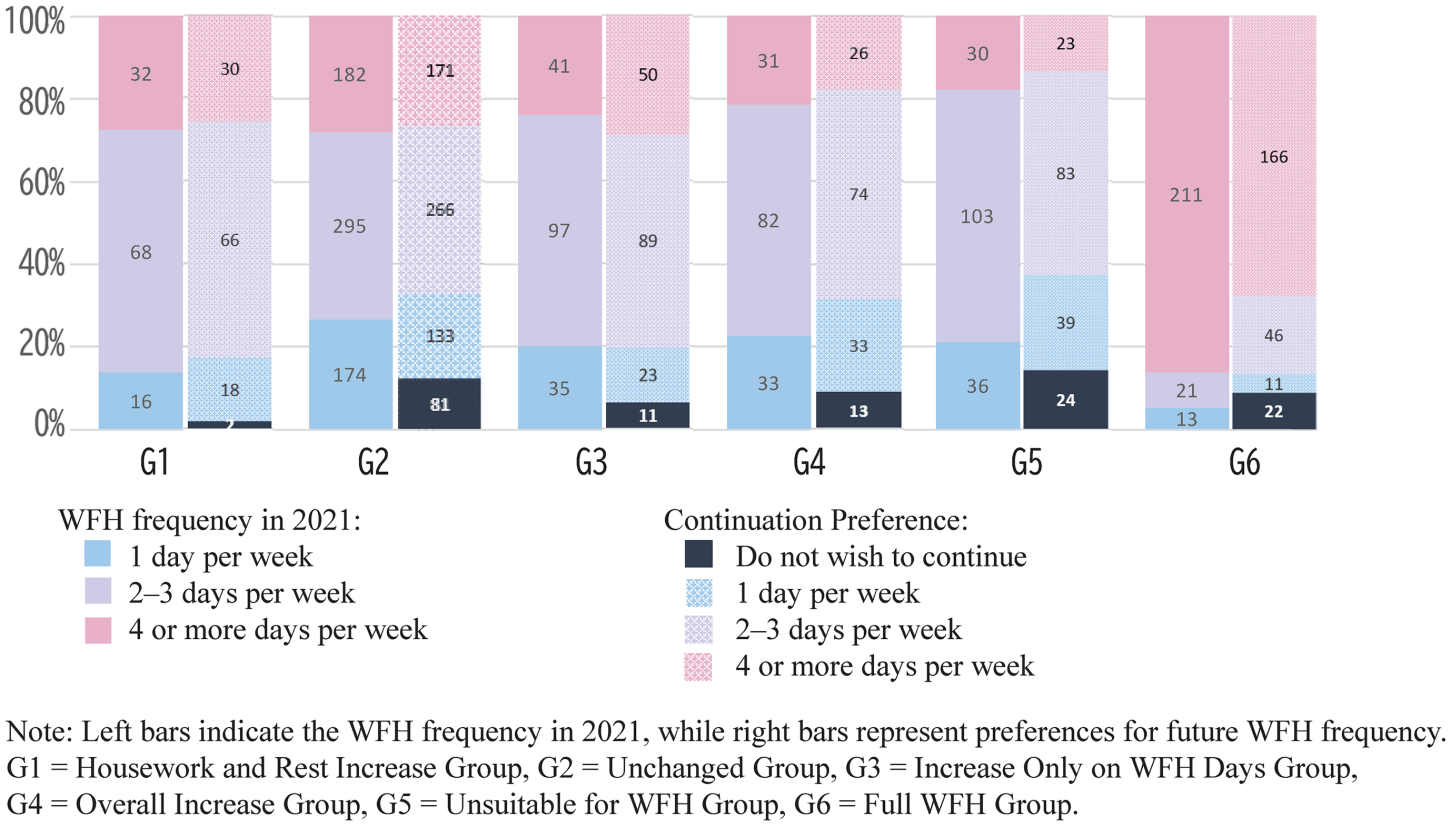
WFH experienced frequency and continuation preference frequency across WFH groups.

Finally, Figure 7 shows the relationship between respondents’ desire to continue teleworking, the frequency of telework they hope to maintain in the future (right bar), and the frequency of telework in 2021 (left bar). Significant differences were found in the desire to continue teleworking (*χ*^2^ = 235.4, *df* = 15, *p* = 0.000, *V* = 0.23). In all groups except the “Full WFH Group,” the most common preference was to continue teleworking 2–3 days per week. This trend aligns with the frequency with which respondents most frequently telework. Overall, 50% of respondents (and 65% when combining those who preferred 2–3 days and those who preferred 4 or more days per week) indicated a desire to continue teleworking at the same frequency they were currently doing. Meanwhile, 10% of respondents indicated they did not want to continue teleworking, with the highest percentages found in the “Unsuitable for WFH Group” and the “Unchanged Group.” Conversely, the “Housework and Rest Increase Group” had the smallest percentage of respondents who did not want to continue teleworking. The increase in housework and rest time on WFH days appears to have enhanced the satisfaction of teleworkers, contributing to their desire to continue teleworking.

## 5 Discussion

Regarding changes in daily life time, respondents could be divided into two groups: those whose leisure and rest time increased only on their WFH days during the telework period, and those whose daily life time increased even on office days. While most respondents experienced no change in daily life time on office days, and decreases were rare, some decreases were observed only in the “Increase Only on WFH Days Group” and the “Unsuitable for WFH Group.” Additionally, there were few respondents who reported an increase only in housework or family care; in most cases, respondents experienced an increase in both leisure and rest time, suggesting that the increase in daily life time contributed to an improvement in their quality of life. Notably, in the “Housework and Rest Increase Group” and the “Overall Increase Group,” many respondents reported an increase in rest time, including time spent with family, even on office days during the telework period. This suggests that even hybrid, low-frequency telework can contribute to an overall improvement in workers’ wellbeing. On the other hand, the “Increase Only on WFH Days Group” showed an increase in daily life time only on WFH days. Respondents in this group were the second most likely to want to continue teleworking four or more days per week, after those in the “Full WFH Group.” Many in this group also expressed a desire to telework more frequently than they currently do, indicating that their wellbeing improved primarily on WFH days with increasing of leisure and rest time rather than across the entire telework period. Additionally, even in the “Unsuitable for WFH Group,” a high percentage of respondents preferred to continue teleworking at a low frequency. They viewed the increase in daily life time on WFH days positively despite experiencing a decrease in work productivity. This suggests that they are seeking an optimal balance between work and life that takes into account both office and WFH days. Excluding the “Unsuitable for WFH Group,” there were no significant declines in work productivity on WFH days across the other groups, indicating that telework may contribute to both improved productivity and increased job satisfaction, thereby enhancing individual wellbeing. However, while there were some group differences in the amount of time spent on activities contributing to self-fulfillment, such as learning and exercising, the increases were relatively small compared to other time use categories. Given that self-fulfillment is considered an important aspect of subjective wellbeing (Diener, 1984; Eid and Larsen, 2008), further examination of time allocation in daily life is warranted.

This study also highlights the limitations of telework as a universally applicable solution, particularly by identifying a group of workers who found hybrid telework increased their working hours, reduced sleep time and decreased productivity on office workdays. These findings align with prior research, which has pointed to communication-related challenges as a significant issue in telework settings (Kniffin et al., 2021; Mun et al., 2022; Yang et al., 2022). Additionally, these results can be interpreted through the lens of the JD-R theory, which explains how job performance and wellbeing are shaped by the balance between work demands and available resources (Bakker and Demerouti, 2017, 2007). For these workers, increased demands—such as increased workload and the lack of clear boundaries between work and rest—may have exceeded the resources available to mitigate stress, such as autonomy or support from colleagues. The challenges observed in both formal and informal communication further suggest that limited resources, like collaborative opportunities or responsive feedback, exacerbated these difficulties. Addressing these gaps by enhancing job resources, such as autonomy, effective communication systems, and task-oriented support, aligns with the JD-R framework and could improve telework effectiveness. Our study found that formal task-oriented communication about work and informal interactions among colleagues did not differ substantially. This suggests that individuals who face communication barriers often experience challenges across both forms of communication. Furthermore, we observed that workers with higher levels of agreeableness appeared to experience greater challenges in communication. The correlation between high agreeableness and perceived communication difficulties may be multifaceted. For instance, trait activation theory suggests that personality traits influence job performance, with outcomes varying positively or negatively depending on situational factors (Tett et al., 2021). Firstly, individuals accustomed to face-to-face interactions may have developed unique interpersonal strategies that do not easily transfer to remote settings. Secondly, those who prioritize social interactions in both professional and personal contexts may be particularly sensitive to reduced communication opportunities and the limitations of online tools. Thirdly, highly agreeable individuals, who often demonstrate empathy and concern for others, may experience stress and frustration in online environments where nonverbal cues are more challenging to interpret, leading to potential misunderstandings. Such individuals might also be especially attuned to the general sense of unease surrounding new communication methods or tools, which can lead them to empathize with others’ anxieties and amplify their own stress. This phenomenon aligns with theories suggesting that agreeable and empathetic individuals are more susceptible to emotional contagion in socially stressful contexts, potentially amplifying their stress levels (Barsade et al., 2018; Shu et al., 2017). To address these challenges, a variety of approaches have been adopted, with some organizations adopting a hybrid work model that combines remote and in office work, while others revert to a full return to the office in alignment with their corporate values and strategic objectives. This variety of strategies underscores the diversity of organizational policies aimed at balancing flexibility with the unique challenges of telework. Innovations supporting effective telework include advancements in video conferencing, improvements in communication tools, and systems that facilitate not only task-based interactions but also informal communication within the workplace (Mitchell, 2021; Viererbl et al., 2022; Watanabe et al., 2024). Such measures highlight the need for tailored support systems that help workers balance the demands of communication with the benefits of telework (Metselaar et al., 2023; Raišienė et al., 2020). These findings indicate that while telework and digital transformation hold the potential to foster better social connections, their impact is highly dependent on the contexts in which ICT tools are used and on users’ attitudes. These tools can either exacerbate social isolation and division or enhance connection and create new opportunities for community engagement. Establishing systems that support the optimal use and diffusion of these tools is crucial. Additionally, encouraging social interactions for individuals who may benefit from them could further enrich the telework experience. To maximize the benefits of telework, flexible policies that provide personalized support, including task redistribution, enhanced ICT infrastructure, and clear guidelines for communication, are essential. By addressing diverse job demands and resources, such policies can mitigate negative outcomes and ensure telework contributes to sustainable work practices and employee wellbeing.

The increase in leisure time brought by WFH could impact environmental sustainability, as previous reviews have suggested, through rebound effects (Horner et al., 2016; O’Brien and Yazdani Aliabadi, 2020). For instance, extended relaxation hours might lead to more frequent video streaming or car use, potentially resulting in environmental impacts that outweigh the reductions achieved by decreased commuting. Conversely, our findings highlight that WFH increases time spent with family, suggesting that shared activities within a common space may lower household energy consumption overall. Preferences for personalized settings in lighting and air conditioning indicate that WFH may improve comfort for many individuals, offering control over environmental conditions that might not be available in office spaces (Beckel and Fisher, 2022). Although energy consumption may temporarily rise if offices maintain centralized systems, this presents an opportunity to consider individualized control systems in the workplace. Such adjustments could improve quality of life for office workers while enhancing the environmental benefits of WFH by reducing redundant energy use (Kanamori et al., 2023). These observations underscore the importance of further investigating high-impact activities that affect both wellbeing and environmental outcomes. Flexibility in commuting patterns is one such possibility. While WFH is typically associated with reduced commuting, its environmental benefits may be limited if commuting behavior remains unchanged. However, introducing more flexible commuting schedules could allow workers to avoid peak traffic times, reducing congestion, thereby lowering both emissions, and stress levels (Moglia et al., 2021). Flexibility not only mitigates environmental burdens but also enhances personal wellbeing by reducing the strain of daily travel. Furthermore, extended hours of video conferencing add to environmental loads. This highlights the importance of fostering workplace environments that emphasize worker autonomy, enabling them to manage their tasks independently rather than requiring frequent check-ins, which may help reduce ICT-related environmental impacts (Aoki et al., 2023b). Framing WFH as a flexible, autonomy-driven work style could enhance both individual wellbeing and environmental sustainability. Realizing these benefits will require cultivating an organizational culture and social systems that support flexible, context-sensitive telework arrangements tailored to individual needs, maximizing their potential to foster wellbeing and environmental responsibility. Such support not only facilitates productivity but also balances social and environmental responsibilities, enabling telework to contribute to both personal wellbeing and broader sustainability goals.

One limitation of this survey is that the time changes were self-reported by respondents in a subjective and relative manner, making absolute comparisons between respondents inappropriate. This study focused on individual perceptions of change, but future research could benefit from using ICT tools to collect objective data while combining it with subjective evaluations. This approach could reduce the burden on respondents while enabling the development of methods for more detailed data collection. Additionally, given that the effects of the COVID-19 pandemic were still strongly influencing society at the time of this study, we were unable to directly analyze the impact of telework on subjective wellbeing. To deepen our understanding of the factors contributing to improvements in quality of life, it would be useful to examine the relationship between actual changes and wellbeing outcomes and the long-term effects of telework.

## 6 Conclusion

This study investigated changes in both work and lifestyle during the telework period, focusing on the differences between WFH days and office days, as well as the combined effects of these changes. We categorized respondents into distinct groups based on changes in their daily life time and work conditions. Specifically, we named these groups according to the primary area of increased time allocation in daily life: the “Housework and Rest Increase Group” (G1), the “Unchanged Group” (G2), the “Increase Only on WFH Days Group” (G3), and the “Overall Increase Group” (G4). Meanwhile, we named the remaining groups based on changes in work efficiency between WFH and office days: the “Unsuitable for WFH Group” (G5) and the “Full WFH Group” (G6). The largest group was the “Unchanged Group,” but for most respondents, their daily life time on WFH days increased during the telework period. On office days, there was a tendency for no change, but the “Housework and Rest Increase Group” and the “Overall Increase Group” showed an increase in daily life time. These findings suggest that telework led to an increase in daily life time, particularly in leisure and rest time, improving the wellbeing of many workers. Moreover, 90% of respondents expressed a desire to continue teleworking, with the most popular preference being 2–3 days per week. The groups that experienced an increase in daily life time on WFH days were also more likely to express a desire to continue teleworking. This implies that telework contributed to enhancing workers’ wellbeing by allowing for more time to engage in daily activities. However, in the “Unsuitable for WFH Group,” respondents reported a decrease in daily life time on office days, an increase in workload, and difficulties with work-related communication. These findings highlight the need for flexible support tailored to individual jobs and personalities, including identifying tasks suitable for telework, redistributing work on office days, and providing support for both work-related and informal communication.

In our efforts to achieve a sustainable society, it is vital to recognize the wide-ranging effects of telework on not only work styles but also lifestyles. Moving forward, it will be important to explore work styles that balance individual wellbeing with broader societal goals, such as reducing environmental impact, improving productivity, and enhancing social inclusivity.

## Data Availability

The datasets presented in this article are not readily available because the original contributions presented in the study are included in the article/Supplementary material, further inquiries can be directed to the corresponding author. Requests to access the datasets should be directed to Eri Aoki, aoki@chikyu.ac.jp.
