# Supplementary material for: Changes and their effects on working and daily life time use allocation between work-from-home and office work days during the telework period: insights from the survey in Japan
Source: Front Sociol. 2025 Feb 4;10:1534548. doi: 10.3389/fsoc.2025.1534548 (PMC11832714; doi:10.3389/fsoc.2025.1534548)
Supplement: Supplementary file 1 [file Data_Sheet_1.PDF]

Supplementary Material

Changes and their Effects on Working and Daily Life Time Use Allocation between Work-from-home and Office Work Days during the Telework Period: Insights from the Survey in Japan

Eri Aoki*, Ai Hiramatsu, and Keisuke Hanaki

*** Correspondence:** Eri Aoki: aoki@chikyu.ac.jp

1 Contents of the Questionnaire Survey

1.1 The working situation of telework: WFH frequency before the COVID-19 pandemic, during the survey period, and desired frequency in the future.

Q. During this year (2021), did you regularly (for approximately one month or longer) engage in telework from home at least one day per week?

Please select the option below that best describes how frequently you worked from home during the period when you engaged in telework most consistently over the longest duration:

1. I did not engage in regular telework.
2. During the longest period, I worked from home an average of 1 day per week.
3. During the longest period, I worked from home an average of 2 days per week.
4. During the longest period, I worked from home an average of 3 days per week.
5. During the longest period, I worked from home an average of 4 days per week.
6. During the longest period, I worked from home an average of 5 or more days per week.

Note: For the purposes of this survey, "working from home" refers to performing work duties at home using a telecommunications network. It does not include work conducted at other locations.

Q. In 2019 and 2020, did you regularly (for approximately one month or longer) engage in telework from home at least one day per week?

Please select all the periods during which you engaged in telework from home:

1. 2019 (before the COVID-19 pandemic)
2. Around April to June 2020 (during the first state of emergency)
3. Around November 2020
4. I did not engage in regular telework during any of the above periods.

Q. If you were to continue working from home after the COVID-19 pandemic subsides, how many days per week would you prefer to work from home?

Please select one option:

1. I do not wish to continue teleworking.
2. I would prefer to work from home approximately 1 day per week.
3. I would prefer to work from home approximately 2 days per week.
4. I would prefer to work from home approximately 3 days per week.
5. I would prefer to work from home approximately 4 days per week.
6. I would prefer to work from home 5 or more days per week.

1.2 The working situation on WFH and office days during the WFH period, including autonomy, workload, productivity, and working hours

Q. During the telework period, on average, how many days per week did you commute to your regular workplace?

Please select one option:

1. I did not work at my regular workplace.
2. I worked at my regular workplace approximately 1 day per week.
3. I worked at my regular workplace approximately 2 days per week.
4. I worked at my regular workplace approximately 3 days per week.
5. I worked at my regular workplace approximately 4 days per week.
6. I worked at my regular workplace 5 or more days per week.

Q. How did you manage your work hours and personal activities while teleworking?

Please select the option that best describes your situation:

1. Working hours were determined by the employer, and personal activities during those hours were completely prohibited and supervised.
2. Working hours were determined by the employer, but some flexibility in personal activities was allowed.
3. Working hours were self-determined, but personal activities during those hours were completely prohibited and supervised.
4. Working hours were self-determined, and some flexibility in personal activities was allowed.
5. Both working hours and personal activities were entirely at my own discretion.

Q. Based on your experience with telework, please select the option that best reflects your thoughts about work and life on WFH days.

Note: Please respond based on your current thoughts about telework, rather than whether each statement matched your actual situation.

(Select one option for each item.)

	Completely disagree	Disagree	Slightly disagree	Slightly agree	Agree	Completely agree
1. I can concentrate on work without interruptions.	☑	☑	☑	☑	☑	☑
2. There are no problems with the work environment.	☑	☑	☑	☑	☑	☑
3. It is desirable to have autonomy over how I perform my work.	☑	☑	☑	☑	☑	☑
4. Additional work or effort is required due to telework.	☑	☑	☑	☑	☑	☑
5. Privacy is a concern.	☑	☑	☑	☑	☑	☑
6. I must personally pay for work-related expenses.	☑	☑	☑	☑	☑	☑
7. My work content is unsuitable for telework.	☑	☑	☑	☑	☑	☑
8. Switching between work and personal time is difficult.	☑	☑	☑	☑	☑	☑
9. Interaction with colleagues or others involved in my work has decreased.	☑	☑	☑	☑	☑	☑
10. Communicating about work is challenging.	☑	☑	☑	☑	☑	☑

(For respondents who did not have office days during the telework period)

Q. Please compare the following aspects of your situation on WFH days during the telework period with the time before the pandemic when you primarily commuted to the office.

(Select one option for each item.)

	Increased	Slightly increased	No change	Slightly decreased	Decreased
1. The amount of work you perform	☑	☑	☑	☑	☑
2. Work productivity	☑	☑	☑	☑	☑
3. Total working hours (excluding commuting time)	☑	☑	☑	☑	☑
4. Hours of sleep	☑	☑	☑	☑	☑

(For respondents who had office days during the telework period)

Q. Please compare the following aspects of your situation during the telework period with the time before the pandemic when you primarily commuted to the office.

(Select one option for each item.)

	Increased	Slightly increased	No change	Slightly decreased	Decreased
1. Amount of work performed on **WFH days**	☑	☑	☑	☑	☑
2. Work productivity on **WFH days**	☑	☑	☑	☑	☑
3. Total working hours on **WFH days** (excluding commuting time)	☑	☑	☑	☑	☑
4. Hours of sleep on **WFH days**	☑	☑	☑	☑	☑
5. Amount of work performed on **office days**	☑	☑	☑	☑	☑
6. Work productivity on **office days**	☑	☑	☑	☑	☑
7. Total working hours on **office days** (excluding commuting time)	☑	☑	☑	☑	☑
8. Hours of sleep on **office days**	☑	☑	☑	☑	☑

1.3 The daily life situation on WFH and office days during the WFH period, focusing on changes in daily life time

Q. Please compare the time you spend on each activity during **WFH days** with the time spent during the period when you primarily commuted to the office.

(Select one option for each item.)

	I didn't do it before and don't do it now	I started doing it	It increased	No change	It decreased
1. Time for Housework	☑	☑	☑	☑	☑
2. Time for Shopping for Food and Daily Necessities	☑	☑	☑	☑	☑
3. Time for Childcare (*)	☑	☑	☑	☑	☑
4. Time for Providing Care and Nursing (*)	☑	☑	☑	☑	☑
5. Time for Relaxation	☑	☑	☑	☑	☑
6. Time Spent with Family and Cohabitants	☑	☑	☑	☑	☑
7. Time for Hobbies and Favorite Activities	☑	☑	☑	☑	☑
8. Time for Exercise (Including Light Exercise, Such as Walking)	☑	☑	☑	☑	☑
9. Time for Personal Study (Including Pursuing Certifications, etc.)	☑	☑	☑	☑	☑

Note: (*) Includes care for non-cohabiting relatives and others.

(For respondents who had office days during the telework period)

Q. During **Office days** within the telework period, please compare the time you spend on each activity with the time spent during the period when you primarily commuted to the office.

(Select one option for each item.)

	I didn't do it before and don't do it now	I started doing it	It increased	No change	It decreased
1. Time for Housework	☑	☑	☑	☑	☑
2. Time for Shopping for Food and Daily Necessities	☑	☑	☑	☑	☑

3. Time for Childcare (*)	☑	☑	☑	☑	☑
4. Time for Providing Care and Nursing (*)	☑	☑	☑	☑	☑
5. Time for Relaxation	☑	☑	☑	☑	☑
6. Time Spent with Family and Cohabitants	☑	☑	☑	☑	☑
7. Time for Hobbies and Favorite Activities	☑	☑	☑	☑	☑
8. Time for Exercise (Including Light Exercise, Such as Walking)	☑	☑	☑	☑	☑
9. Time for Personal Study (Including Pursuing Certifications, etc.)	☑	☑	☑	☑	☑

Note: (*) Includes care for non-cohabiting relatives and others.

1.4 Personal traits: The Japanese version of the Ten Item Personality Inventory (TIPI-J; Oshio et al., 2012)

Q. Please select the option that best describes how well each statement applies to you. Consider the entire statement comprehensively and evaluate how much it resonates with your self-perception.

I see myself as …

	Strongly disagree<br>1	Disagree somewhat<br>2	Slightly disagree<br>3	Neither agree nor disagree<br>4	Slightly agree<br>5	Agree somewhat<br>6	Strongly agree<br>7
1. Someone who is active and outgoing.	☑	☑	☑	☑	☑	☑	☑
2. Someone who tends to hold grudges and easily causes conflicts.	☑	☑	☑	☑	☑	☑	☑
3. Someone who is diligent and self-disciplined.	☑	☑	☑	☑	☑	☑	☑

4. Someone who is anxious and easily flustered.	☑	☑	☑	☑	☑	☑	☑
5. Someone who likes new experiences and has unconventional ideas.	☑	☑	☑	☑	☑	☑	☑
6. Someone who is reserved and quiet.	☑	☑	☑	☑	☑	☑	☑
7. Someone who is considerate and kind to others.	☑	☑	☑	☑	☑	☑	☑
8. Someone who is careless and inattentive.	☑	☑	☑	☑	☑	☑	☑
9. Someone who is calm and emotionally stable.	☑	☑	☑	☑	☑	☑	☑
10. Someone who lacks creativity and is rather ordinary.	☑	☑	☑	☑	☑	☑	☑
